# One-Step Method for Direct Acrylation of Vegetable Oils: A Biobased Material for 3D Printing

**DOI:** 10.3390/polym15143136

**Published:** 2023-07-24

**Authors:** Cristian Mendes-Felipe, Igor Isusi, Olga Gómez-Jiménez-Aberasturi, Soraya Prieto-Fernandez, Leire Ruiz-Rubio, Marco Sangermano, José Luis Vilas-Vilela

**Affiliations:** 1 BCMaterials, Basque Center for Materials, Applications and Nanostructures, UPV/EHU Science Park, 48940 Leioa, Spain; joseluis.vilas@ehu.eus; 2 Department of Applied Science and Technology (DISAT), Politecnico di Torino, 10129 Torino, Italy; marco.sangermano@polito.it; 3 Macromolecular Chemistry Group (LABQUIMAC), Department of Physical Chemistry, Faculty of Science and Technology, University of the Basque Country (UPV/EHU), 48940 Leioa, Spain; 4 TECNALIA, Basque Research and Technology Alliance (BRTA), Parque Tecnológico de Álava, Leonardo Da Vinci 11, 01510 Minano, Spain; olga.gomez@tecnalia.com (O.G.-J.-A.); soraya.prieto@tecnalia.com (S.P.-F.)

**Keywords:** soybean oil, one-step reaction, biobased polymers, UV-curable, 3D printing

## Abstract

The substitution of fossil resources by alternatives derived from biomass is a reality that is taking on a growing relevance in the chemical and energy industries. In this sense, fats, oils, and their derived products have become indispensable inputs due to their broad functional attributes, stable price and sustainable character. Acrylated vegetable oils are considered to be very versatile materials for very broad applications (such as in adhesives, coatings or inks) since, in the presence of photoinitiators, they can be polymerized by means of UV-initiated free radical polymerizations. The usual process for the synthesis of acrylate vegetable oils consists in reacting epoxidized oils derivatives with acrylic acid. Here, the influence of different catalysts on the activity and selectivity of the process of acrylation of epoxidized soybean oil is studied. In addition, a novel one-step method for direct acrylation of vegetable oils is also explored. This new approach advantageously uses the original vegetable resource and eliminates intermediate reactions, thus being more environmentally efficient. This study offers a simple and low-cost option for synthesizing a biomass-derived monomer and studies the potential for the 3D printing of complex structures via digital light processing (DLP) 3D printing of the thus-obtained novel sustainable formulations.

## 1. Introduction

The modern world is constantly changing in order to be able to satisfy all requirements at both technological and industrial levels. In this sense, the 4th industrial revolution, also called Industry 4.0, began some years ago as a revolution that utilizes information technology in the manufacturing sector to enable smart manufacturing [1], highlighting additive manufacturing (AM) as being among the most useful process. AM is defined as a process that “makes ‘objects’ from a digital ‘model’ by depositing the constituent material/s in a layer-by-layer manner using digitally controlled and operated material laying tools” and includes eight different processes: binder jetting (BJ), directed energy deposition (DED), material extrusion (ME), material jetting (MJ), powder bed fusion, sheet lamination (SL) and vat photopolymerization (VP). Among these methods, VP needs to be highlighted as it allows the production of large parts with excellent accuracy, surface finish and details [2]. Particularly, digital light processing (DLP) 3D printing is among the most useful techniques of VP due to its basis in the layer-by-layer curing of liquid resin formulations using digital micro-mirror devices (DMD) that induce an instantaneous entire layer polymerization, compared to stereolithography (SLA) methods that cure liquid formulations using single-laser point technology [3]. In addition, as a UV light-induced polymerization technique, DLP presents advantages because it is a fast, economic and nontoxic method that works at room temperature, with space and energy efficiency, and produces high-resolution patterns compared to the other AM processes [4].

Conversely, the current situation of the planet due to the growth of the greenhouse effect, climate change, and fossil fuel resource depletion, requires the adoption of a circular economy (CE) strategy in which the production process relies on the principles of sustainability, such as green purchasing, reusing, recycling, and remanufacturing [5]. Furthermore, the COVID-19 pandemic situation has drastically increased the consumption of disposable polymer-based materials [6]. Thus, the use of materials obtained from sustainable sources becomes more important year by year. Among the different possible options of renewable materials, terpenes, carbohydrates, and vegetable oils are the most used monomers for the plastic production [7]. In particular, oils obtained from vegetables, such as cardanol [8], are important renewable feedstocks for the synthesis of UV-curable biobased materials because of their low cost in comparison to other biomass material, as well as their availability and biodegradability [9].

Here, soybean oil (SO) stands out as one of the oils presenting the largest global production volumes (together with palm and rapeseed oil) with an economical price for large-scale uses [10]. Additionally, it presents low toxicity, biocompatibility, inherent biodegradability, high thermal stability, and good mechanical properties, and can be easily combined with fillers to make functional materials [11].

However, AM processes, particularly DLP printing, are not optimized for the use of materials obtained from renewable sources. The most promising ones for use as DLP resins are vegetable oils (such as the aforementioned soybean oil) because they have liquid or quasi-liquid states, similar to those of the existing petroleum-based resins, and as they present functional groups along their chains, which are similar to those needed for UV curing, such as the carbon–carbon double bonds of the triglyceride [10,12]. Despite this, it was demonstrated that the reactivity of those groups against UV light in the presence of a photoinitiator was very low [13]. For this reason, several methods have been developed to functionalize vegetable oils, including epoxidation and/or acrylation. Epoxidation is one of the most used methods when photocurable materials for film and coating applications are needed, while acrylation is most appropriately used to obtain 3D printable resins. The epoxidation reaction of vegetable oils has been tested for several oils such as peanut, rapeseed, rose hip, safflower, or camelina oils, among others. This epoxidation consists in reacting internal alkenes of vegetable oils using oxidizing systems to form the epoxy groups. This is typically carried out using one of these four epoxidation methods: in situ with peracids in the presence of an inorganic soluble or supported catalyst; with organic or inorganic peroxides, using transition metals or enzymatic species as catalysts; via halohydrins formation; or using molecular oxygen [14,15,16]. On the other hand, acrylation of vegetable oils has been generally carried out as a two-step process consisting in an initial epoxidation reaction (as explained above), followed by acrylation using acrylic acid. This is the common way of obtaining acrylated epoxidized vegetable oils, such as the ones made of soybean, palm, castor, linseed, or Jatropha seed [17,18,19]. However, in terms of green chemistry (atom economy) and cost-effectiveness, it would be desirable to synthesize acrylated vegetable oils via the direct addition of acrylic acids to the carbon–carbon double bond of the triglyceride as it is prone to capturing electrophiles [20]. In this sense, the direct acrylation reaction has been studied for use in soybean oil [21,22].

In this way, the main objective of this work is to synthesize and characterize acrylated epoxidized soybean oil (AESO) and direct acrylated soybean oil (ASO), and to study their application as suitable formulations for DLP 3D printing, comparing the final mechanical and viscoelastic properties of UV-cured materials.

## 2. Materials and Methods

### 2.1. Materials

Epoxidized soybean oil (ESO) EPOVINSTAB H-800D, with a viscosity of 550–650 cP at 20 °C, was obtained from Hebron SA (La Llagosta, Spain) while soybean oil (SO) was obtained from KM Elite products (Petwoth, UK). The acrylic acid anhydrous (AA), presenting a purity of 99%, was purchased on Fluka (Buchs, Switzerland) and hydroquinone with a purity of 99.5% was acquired from Acros Organics (Hampton, NH, USA). Five different materials were used as catalysts of ESO acrylation: triethylamine (TEA) with a purity of 99% from Fisher Chemical, triphenylphosphine (TPP) with a purity of 99% from Acros Organics, and three different chromium salts, namely, chromium (III) 2-ethylhexanoate at 50% purity in 2-ethylhexanoic acid (Cr(EH)_3_-EHXA) from Alfa Aesar (Kandel, Germany), chromium (III) 2,4-pentanedionate (Cr(acac)_3_) with a purity of 97% from Alfa Aesar, and chromium (III) 2-ethylhexanoate (Cr(EH)_3_-MO) at 70% in mineral oil from VladaChem (Malsch, Germany). In the case of acrylation of SO, boron trifluoride (BF_3_) at 48% purity in diethyl ether (Et_2_O), obtained from Acros Organics, was used as a catalyst. Ethyl acetate with a purity of 99.8% was obtained from Fisher Scientific (Hampton, NH, USA) and sodium sulphate anhydrous with a purity of 99.0% was purchased from Sigma Aldrich (Darmstadt, Germany). Deuterated chloroform, presenting a purity of 99.8%, was acquired from Sigma Aldrich and used as a solvent in nuclear magnetic resonance experiments.

Phenylbis(2,4,6-trimethylbenzoyl) phosphine oxide with a purity of 98% was purchased from Sigma Aldrich and used as a photoinitiator. Ethanol (EtOH) purchased from Merck (Darmstadt, Germany) with 96% of purity was used as a sample cleaner. Poly(ethyleneglycol) di-acrylate (PEGDA) of a molecular weight of 575 g/mol from Sigma Aldrich (Darmstadt, Germany) was used as a sacrificial material during the 3D printing process.

### 2.2. Synthesis of Acrylated Epoxidized Soybean Oil (AESO)

AESO was obtained by opening the oxirane groups present in the fatty chains of commercial epoxidized oils with acrylic acid. Reactions were carried out in a batch mode using mechanically stirred glass reactors with a 1.1 molar excess from acrylic/epoxide groups. Temperature was maintained at 80 °C for 6 h. In total, 1% (*w*/*w*) hydroquinone was employed as a polymerization inhibitor. The efficiency of different nucleophilic catalysts was evaluated using the same operational conditions in order to select the most appropriate catalysts for use in the process. TEA and TPP were selected as two of the most common catalysts used in acrylation reactions [18], together with three salts of chromium (III).

Products were dissolved with ethyl acetate and washed several times with brine until the complete removal of the rest of the catalyst, hydroquinone, and acrylic acid. This process was finished with the determination of a neutral pH for the aqueous phase. On the other hand, the organic phase was collected, dried over sodium sulphate and filtered. Finally, the remaining organic solvent was eliminated via vacuum distillation. The evolution of the acrylation of epoxidized oils was followed by epoxide index and acidity measurements.

### 2.3. Synthesis of Acrylated Soybean Oil (ASO)

Soybean oil (SO) and acrylic acid (AA) were premixed in specific proportions in a glass reactor equipped with a condenser, thermometer, and magnetic stirring. When the temperature achieved 80 °C, BF_3_·Et_2_O was added as a catalyst to the system. The acrylation reactions were maintained for 10 h. The purification of products was carried out following the same protocol as is employed in the acrylation of epoxidized oils.

### 2.4. Characterization of Acrylated Oils by Epoxy Index (E.I.), Acidity Index and Iodine Value

The disappearance of the oxirane groups present in the ESO gives an idea about the evolution of the acrylation reaction and its corresponding conversion. This factor was assessed from the epoxy oxygen content determination, which was performed with the standard AOCS analysis method Cd 9–57.

Conversely, the presence of free acidic species in the reaction mixture was determined via the acidity index according to the norm (UNE-EN-ISO 660 [23]). We determined the free acrylic acid that remained after the reaction process. Finally, the iodine value was measured via the Wijs method [24]. This analysis permitted us to establish the unsaturations present in the soybean oil.

### 2.5. Fourier Transformed Infrared Spectroscopy (FTIR)

Fourier transformed infrared spectroscopy (FTIR) was used to analyze the main chemical groups of the molecular structures of the different oils synthesized. The five different acrylated epoxidized soybean oils (AESO) obtained depending on the catalyst used, as well as the acrylated soybean oil (ASO), were analyzed. In addition, the synthesis reaction of AESO using the chromium (III) 2-ethylhexanoate at 70% in mineral oil (Cr(EH)_3_-MO) catalyst was followed at different times via FTIR, and epoxidized soybean oil (ESO) and soybean oil (SO) were studied in order to compare the initial and final products obtained. A Bruker Alpha Platinum ATR-FTIR spectrophotometer (Billerica, MA, USA) was employed for experimental measures. Spectra were collected at a resolution of 4 cm^−1^, in transmittance mode, within a range of 400 to 4000 cm^−1^. A total of 24 scans were acquired for each analysis.

Furthermore, attenuated total reflectance-Fourier transform infrared spectroscopy (ATR-FTIR) equipment was used to monitor the photopolymerization reaction of AESO and ASO formulations (AESO-L and ASO-L prepared as explained in Section 2.7) by assessing the disappearance of the acrylic double bond peak at 1630 cm^−1^. Samples were measured first in a liquid state (pre-cured) and then a solid state (post-cured) after 90 s irradiation at room temperature and under nitrogen atmospheric conditions using a Dymax ECE 5000 UV lamp (130 mW/cm^2^ of irradiance) (Frankfurt, Germany). For ATR-FTIR, we used a Thermo Scientific Nicolet iS50 FTIR Spectrometer (Waltham, MA, USA) instrument equipped with a diamond crystal ATR accessory. For each measurement, 32 spectra were obtained in the spectral range from 4000 to 600 cm^−1^ at a resolution of 4 cm^−1^. The averaged value after 3 measurements was indicated.

### 2.6. Nuclear Magnetic Resonance (^1^H-NMR)

The chemical structure and the degree of acrylation (AD) of AESO and ASO were determined via NMR spectroscopy. Briefly, ^1^H-NMR spectra were taken in CDCl_3_ on a Bruker Avance 300 MHz spectrometer at 25 °C, employing a polymer concentration of 33 mg/mL for all oils measured. For the quantification of the modification of epoxidized soybean oil (ESO) and soybean oil (SO), the relative area of the hydrogen atoms of a group that does not change during the reaction, i.e., hydrogen atoms of the carbon next to the carbonyl group, was used as a reference for the determination of protons at an acrylate group.

### 2.7. Preparation of 3D Printable Formulations and Solid Samples

Liquid formulations for use as 3D printable materials were prepared by mixing the corresponding oil (AESO for the AESO-L sample and ASO for the ASO-L sample) with a 1 wt.% of photoinitiator (sufficient quantity to ensure photopolymerization in relatively short periods of time [10]). A homogeneous solution is obtained after 30 min of magnetic stirring at 50 °C for AESO material and at room temperature for ASO material. Then, in order to de-gas the liquid formulations, samples were sonicated for 1 h in an ultrasound bath at a controlling temperature above 40 °C.

AESO-L and ASO-L formulations were used to obtain the corresponding solid samples AESO-S and ASO-S, respectively. For solid samples, liquid formulations were added to a silicone mold and cured via illumination with a Dymax ECE 5000-UV lamp for 60 s at 130 mW/cm^2^ of irradiance under a nitrogen atmosphere.

In the case of 3D printing, samples were prepared using the 3D printer Asiga MAX X27 DLP printer (Erfurt, Germany) presenting a diose source emitting at 405 nm, a building volume of 51.8 × 2.92 × 75 mm, and a XY pixel resolution of 27 µm. During the printing process, a sacrificial PEGA base (around 50 µm thickness) was used to easily remove the printed samples. The layer thickness and light intensity were fixed at 50 µm and 40 mW/cm^2^, respectively. Exposure time was selected for each formulation attending to the photorheology results, while the temperature was controlled to be constant at 35 °C. After printing, samples were washed in an ethanol bath under ultrasonication conditions for 5 min and post-cured 3 min under UV light in a robot factory medium-pressure mercury lamp at 12 mW/cm^2^ of irradiance.

### 2.8. Characterization of 3D Printable Formulations

The samples AESO-L and ASO-L were characterized in terms of viscosity and photocuring. Viscosity measurements were performed at 25 °C in a cone–plate geometry CP50-1 (diameter of 25 mm and cone angle of 1°) with a fixed gap of 1 mm using an Anton Paar MCR302 instrument (Ganz, Austria). The viscosity was recorded by varying the shear rate ($\dot{\gamma }$) of the samples from 1000 to 0.01 s^−1^.

In the case of photocuring, both the total carbon–carbon double bond conversion and the reactivity of the liquid formulations were evaluated. For the reactivity, photorheology tests were carried out using an Anton PAAR Modular Compact Rheometer (Physica MCR 302, Graz, Austria) in a parallel plate configuration with a plate distance of 0.3 mm (upper disk diameter of 25 mm and quartz as a bottom disk). The measurements were performed at a constant frequency of 1 Hz and temperature of 25 °C. When the signal stabilized after 60 s, samples were irradiated from the bottom with a UV light of Hamamatsu LC8 lamp at 30 mW/cm^2^ of irradiance. The evolution of both the storage and loss moduli (G′ and G″, respectively) in time was recorded, and the averaged results of each sample after three measurements were presented. For the total C=C double bond conversion, ATR-FTIR technique was used as explained in Section 2.5.

### 2.9. Characterization of Solid Samples

The characterization of AESO-S and ASO-S was performed on samples prepared using a Dymax ECE 5000-UV lamp. Dynamic mechanical thermal analysis (DMTA) was performed on rectangular samples of 20 mm × 7.5 mm × 1 mm using a Tritec 2000 DMA equipment from Triton technology Ltd. (Leicester, UK). Samples were tested by setting a temperature ramp of 2 °C/min at a fixed frequency of 1 Hz with a total displacement of 20 µm in the temperature range of −60 to 120 °C. The apparent crosslinking density (*ν_c_*) of both AESO-S and ASO-S was calculated applying the Equation (1):

(1) $$\nu_{c}=\frac{{E^{\prime }}_{R}}{3RT}$$

where ${E^{\prime }}_{R}$ is the storage modulus at the rubbery plateau (obtained from the graph at a temperature of 50 °C above the glass transition temperature), R is the gas constant, and T the absolute temperature.

To evaluate the mechanical properties of the prepared solid samples (AESO-S and ASO-S), stress–strain tests were performed in specimens that had dimensions of about 5 mm × 50 mm × 1.10 mm with a Metrotec FTM-50 (20 N load cell) (Lezo, Spain) at room temperature and at a deformation rate of 1 mm/min. The Young modulus (E) was obtained by calculating the slope of the linear region and the strain (ε_b_) and stress (σ_b_) at break were provided in a way that takes five replicates into consideration.

Moreover, the 3D-printed samples were also characterized in terms of the quality of the printed materials. Three-dimensional scanning of the printed objects was performed with a E4 3D scanner from 3Shape and a heat map of the difference between the digital file and the digitalization of the real object created by the 3D scanner was reported.

## 3. Results

### 3.1. Synthesis and Characterization of Acrylated Epoxidized Soybean Oil (AESO), Effect of the Catalyst Nature

Acrylated epoxidized soybean oils are commonly synthesized through two consecutive steps. First, the vegetable oil is epoxidized to transform the double bonds of the fatty chains into epoxide functionalities. In the second step, these epoxide groups react with the carboxylic groups of acrylic acid to give the desired product.

Epoxide groups are tensioned and stressed structures which are very prone to reacting with nucleophiles that interact with the electrophile carbon of the C-O bond, making the ring opening easy [25]. This C-O bond is also polarized by electrophilic compounds, such as protic acids, or Lewis acids that can interact with the electron pair of the oxygen. As a result, carboxylic acid reacts with the epoxide group, giving an ester and hydroxyl functionality. As the epoxidation of vegetable oils is a mature industrial process, commercial epoxidized oil was directly reacted to acrylic acid in order to study the second step of the process. Five different catalysts were considered to speed up the acrylation reaction: TEA and TPP had been previously referenced in the bibliography [26]. On the other hand, a commercial chromium (III) complex salt named AMC-2^®^ accelerator had been demonstrated to be effective in the acrylation of epoxides with organic acids [27]. Here, three alternatives to AMC-2^®^ were compared: chromium (III) 2,4-pentanedionate at 97%, chromium (III) 2-ethylhexanoate at 50% in 2-ethyl hexanoic acid, and chromium (III) 2-ethyl hexanoate in 70% in mineral oil. All catalysts were tested in equivalent conditions: 80 °C; a small molar excess of acrylic acid to oxirane oxygen of 1.1:1; 1% hydroquinone to prevent homopolymerization; a molar ratio (MR) of catalyst to epoxidized oil of 1% b (*w*/*w*); and a reaction time of 6 h.

Making a comparison between the different catalysts employed, it was found that the chromium (III) 2-ethyl hexanoate in 70% of mineral oil presented the highest epoxide conversion (98%), followed by the chromium (III) 2-ethylhexanoate 50% in 2-ethyl hexanoic acid. Next, the chromium (III) 2,4-pentanedionate 97% offered a conversion of 69%, and finally, the least active catalysts were triphenylphosphine (68%) and triethylamine (65%).

**Scheme 1:**
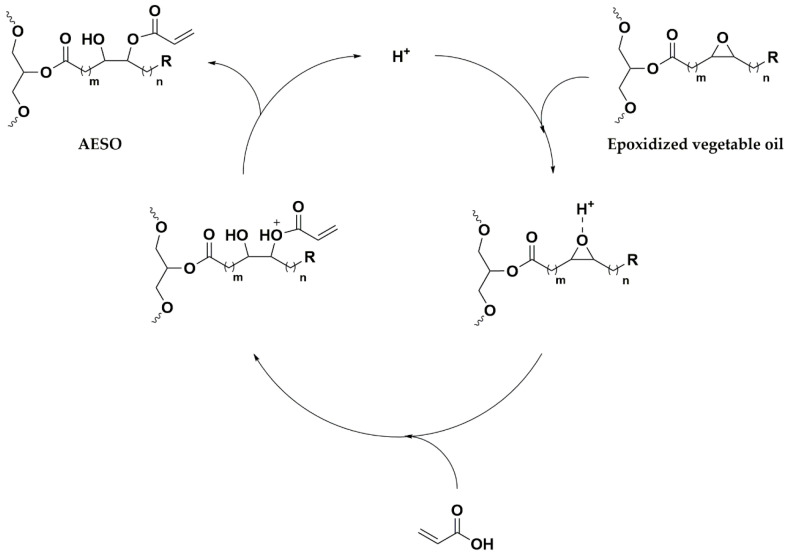
Reaction mechanism of the acrylated epoxidized oil.

As depicted in Scheme 1, the role of the catalyst is related to its acidic/basic properties, as well as its ways of activating the reactant molecules to produce the acrylated oil. In this way, the best catalyst is the one that possesses dual behavior. Ethyl hexanoate and petanedionate anions present basic properties. They abstract a proton from the acrylic acid to form an acrylate anion. On the other hand, Cr (III) is a Lewis acid. It polarizes the C-O bond in the epoxide ring (STEP 1), making the carbon atom more electrophilic, thus providing the attack of the nucleophile. It results in easier epoxide ring opening (STEP 2). Finally, the bases transfer the proton to the previously formed anion, giving acrylated oil (STEP 3). TPP and TEA are exclusively basic catalysts, meaning that their efficiency in the reaction is related to their basicity degree.

Although the conversions are quite similar using chromium (III) 2-ethyl hexanoate diluted in either mineral oil or in 2-ethyl hexanoic acid, it is necessary to consider that in the second case, the solvent can compete with acrylic acid in terms of opening the epoxide rings, thus lowering the selectivity and yield of the acrylated oil.

**Figure 1:**
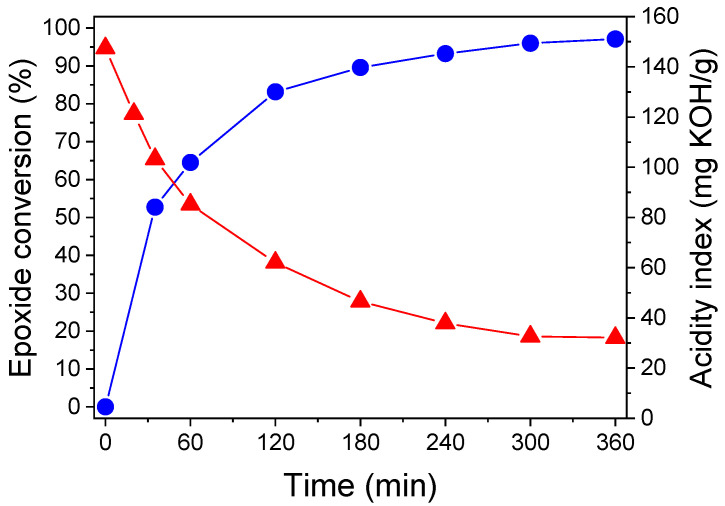
Evolution of the acrylation reaction using chromium (III) 2-ethyl hexanoate in 70% in mineral oil. (● epoxide conversion %; ▲ acidity index (mg KOH/g)). Operational conditions: temperature = 80 °C; molar ratio AA/epox. = 1.1:1; 1% (*w*/*w*) hydroquinone.

The use of chromium (III) 2-ethyl hexanoate in 70% in mineral oil allowed the completion of the acrylation reaction in equivalent operational conditions to those of the AMC-2^®^ catalyst. The epoxide conversion of the reaction, which was quantified via the disappearance of the oxirane groups of the vegetable oil at the beginning and the end of the reaction, indicated it was concluded after 5 h. On the other hand, the acrylic acid content in the reaction media was quantified via the acidity index in the product. It was stable at a value corresponding to 32 mg KOH/g after 5 h of reaction, indicating that all the reactant needed had already been consumed, as can be observed in Figure 1.

**Figure 2:**
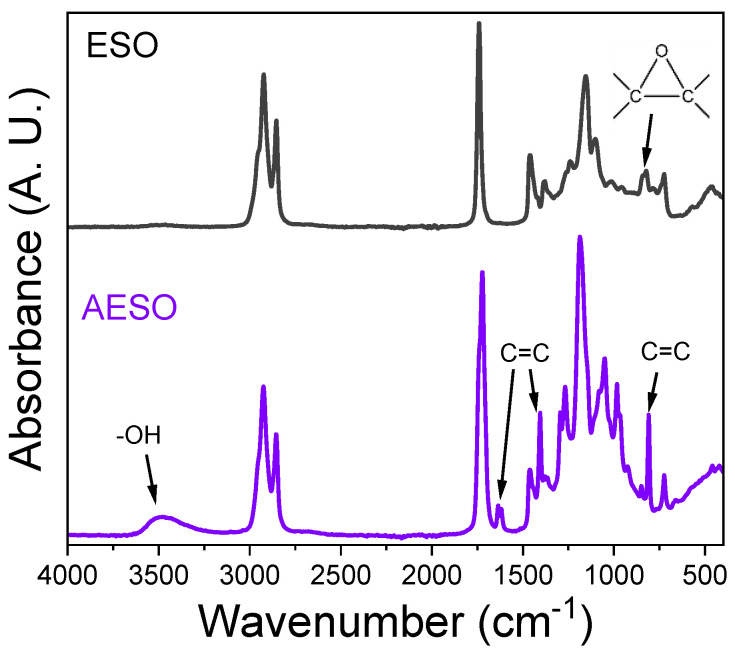
FTIR spectra of ESO and AESO.

According to the FTIR spectra (Figure 2), the product obtained was AESO. The complete disappearance of the peak at 823–830 cm^−1^ indicates that epoxide groups react to form other functionalities. Instead, there is an absorption band of 3400–3500 cm^−1^, associated with the band of –OH. The vinyl group (=C–H) from the acrylate group appears at 3100 cm^−1^. The signal of double bonds (C=C) from the acrylate pendant groups is found at 1650, 1617, and 1406 cm^−1^. It can be also observed a peak at 810 cm^−1,^ a phenemenon which is related to the acrylate out-of-plane deformation [28]. Finally, other peaks, such as the one at 750 cm^−1^, correspond to the band of several continuous methylenes (-CH_2_CH_2_CH_2_CH_2_-) from fatty acid chains.

**Figure 3:**
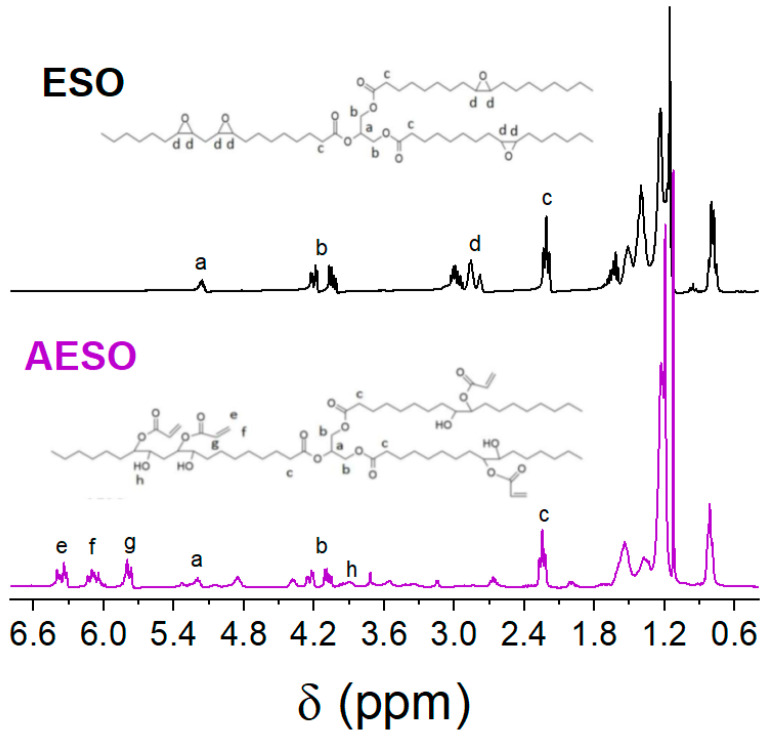
^1^H-NMR spectra of ESO and AESO.

Figure 3 shows the ^1^H-NMR spectra for ESO and AESO and gives their proton attributions. The epoxidation of the vegetable oils was evidenced by the disappearance of the peaks at 5.2–5.5 ppm, which indicated that the vinylic unsaturated hydrogens of the SO were transformed into epoxy groups. These signals appeared again in the AESO sample due to the presence of new acrylic unsaturations. The signals a (δ = 5.25 ppm), b (δ = 4.1 and 4.2 ppm) and c (δ = 2.4 ppm) appeared in both spectra ESO and AESO since they corresponded to the protons of the tri-ester group, which did not suffer modifications during the acrylation reaction. On the other hand, a peak that corresponded to the epoxy functionalities appeared at δ = 2.9 ppm (d) and disappeared in the AESO sample. In the same way, the peak at δ = 1.45 ppm that corresponded to -CH hydrogens adjacent to epoxy groups disappeared in the acrylated sample. The signals at δ = 6.5, 6.2 and 5.9 ppm (e, f, g) corresponded to the cis, trans and gemminal hydrogens with respect to acrylate ester groups. They confirmed the presence of acrylic functionalities in the AESO product.

### 3.2. Synthesis and Characterization of Directly Acrylated Soybean Oil (ASO)

**Scheme 2:**
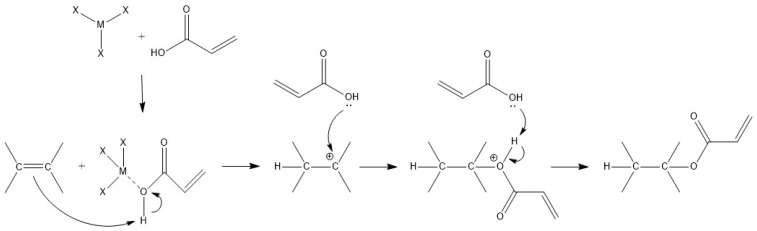
Reaction mechanism of the direct acrylation of double bonds.

The double bonds present in the vegetable oils can directly react with acrylic acid through a one-step process without their intermediate transformation into epoxide rings. However, this reaction requires a strongly acidic catalyst [21] and different operational conditions to activate double bonds of the fatty chains with low activity. Additionally, the polymerization of acrylic acid should be avoided at the same time. Zhang et al. [20] propose a plausible mechanism that explains the direct acrylation of SO with AA, as can be seen in Scheme 2.

**Figure 4:**
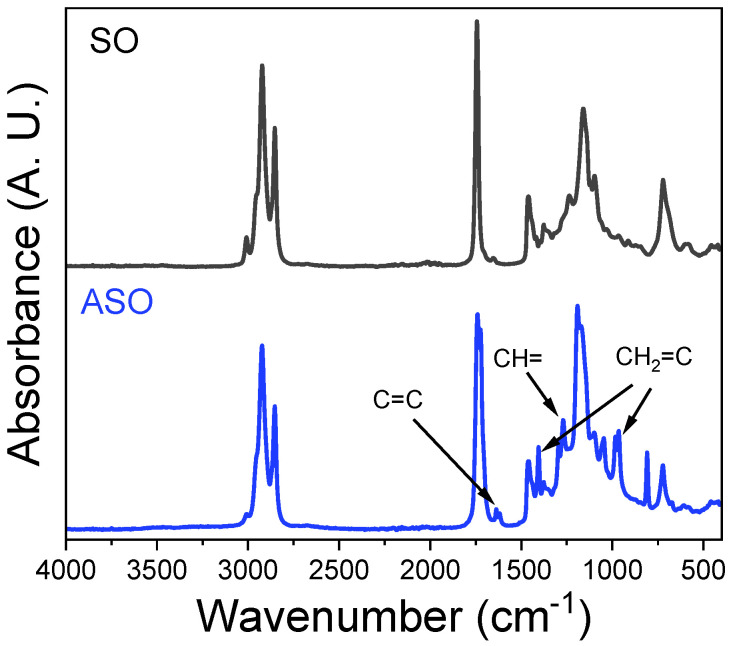
FTIR spectra of SO and ASO.

First, the SO unsaturation degree was determined via the iodine index analysis (Wij’s method). Then, BF_3_OEt_2_ was employed as an efficient Lewis acid capable of inserting the acrylic acid molecules into the double bonds of the fatty chains. Overall, 0.1 equiv of this catalyst were used since higher amounts promoted oligomerization reactions between acrylic acid and SO. A molar ratio between the double bonds and acrylic acid of 1:6 was employed to increase the contact between the reactive groups. It was observed that higher AA amounts were not conducted in higher conversions. However, this was supposedly an important cost of obtaining a valuable reactant and had more drawbacks in the purification stage. The temperature was maintained at 80 °C for 10 h. Increasing the temperature value permitted us to achieve higher conversions faster. However, secondary reactions also appeared. Hydroquinone was used to prevent the polymerization of acrylic acid. However, in this case, the concentration was lowered to 0.25% (*w*/*w*) because hydroquinone acrylate can formed in the presence of this strongly acidic catalyst. The FTIR spectra of ASO indicated some differences from the corresponding one of AESO, as can be seen in Figure 4.

The C=C vibration of the acrylate groups in ASO appeared at 1637 and 1619 cm^−1^ and was considerably more intense than that seen at the corresponding peak at 1652 cm^−1^. This could be attributed to the C=C vibration in SO. Another two peaks appeared at 1400 and 960 cm^−1^, respectively, corresponding to the scissoring vibration and the rocking vibration of CH_2_ in CH_2_=C of the acrylate group. Finally, the two peaks at 1296 and 1272 cm^−1^ corresponded to the vibration of CH in acrylate CH= [28]. The most relevant difference between AESO and ASO was evidenced by the absence of the peak 3400–3500 cm^−1^ that corresponded to the -OH groups of AESO since no hydroxyl functionalities were generated by the direct insertion of acrylic acid into SO.

**Figure 5:**
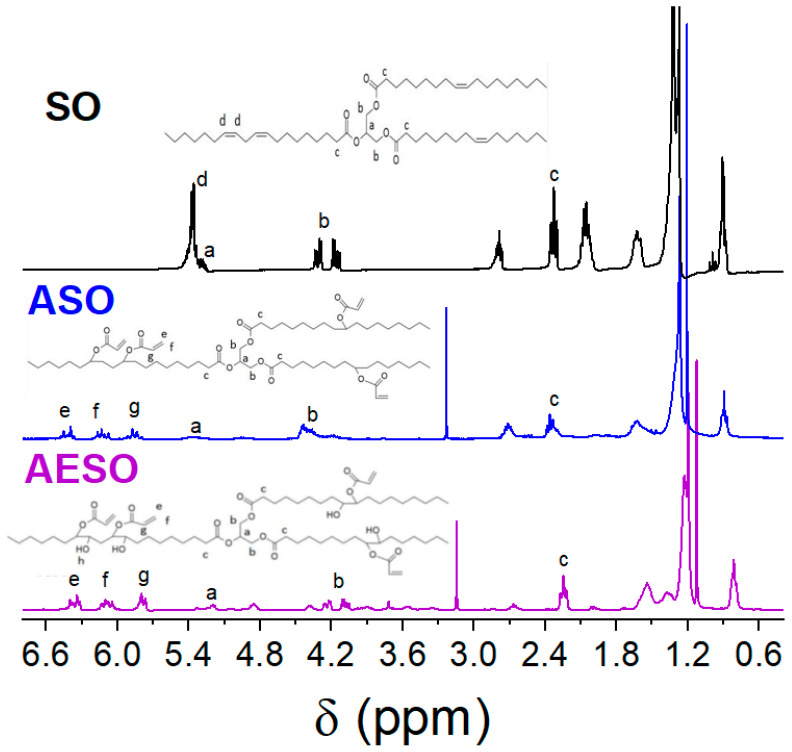
^1^H-NMR spectra of SO, ASO and AESO.

Regarding the ^1^H-NMR results (Figure 5), whereas in the synthesis of AESO the signals corresponding to the epoxide groups of ESO disappeared (δ = 2.9 ppm), the direct acrylation of SO to give ASO showed a partial reaction conversion since the signals of the double bonds of the triglyceride chains decreased (δ = 5.4 ppm) but remained in the product. Referencing Zhang et al. [20], an average rate of 1.44 acrylate molecules per triglyceride was inserted, which was inferior to the percentage achieved in the acrylation of epoxidized oils.

### 3.3. Characterization of 3D Printable Formulations

**Figure 6:**
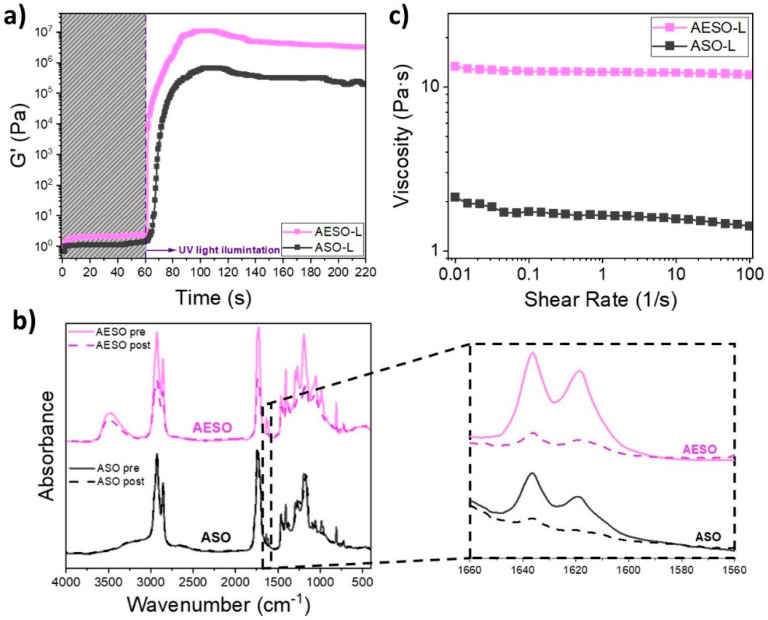
Photorheology curves (**a**), FTIR spectra before (pre-) and after (post-) irradiation with UV-light showing the double bond conversion (**b**) and viscosity as a function of shear rate at 25 °C (**c**) of AESO and ASO formulations.

Once the starting polymeric precursors AESO and ASO have been prepared and characterized, they are mixed with a 1 wt.% of photoinitiator in order to obtain the photocurable formulations. Mainly, resins for use in DLP printing need to be characterized in a way that attends to photoreactivity and viscosity [29]. The photopolymerization processability of AESO-L and ASO-L formulations was evaluated via real-time photorheology, measuring the evolution of both storage and loss moduli (G′ and G″, respectively), while samples were irradiated with UV-light at 405 nm. Thus, G′ values as a function of time (t) are reported in Figure 6a, while values of gel time (t_gel_) calculated as the time at which the crossover of both moduli and the curing rate (ΔG′/Δt) occurs, measured as the slope of the G′ curves in the initial irradiation times, are indicated in Table 1.

The photorheology curves show that both acrylated oils present similar behavior and offer a fast cure by UV light, as can be identified by the large slopes of both curves and the short time that they take to arrive at the plateau. When formulations are irradiated, the photolysis of the initiator generates radicals that rapidly react with the double bonds of the acrylated oils, thus generating a crosslinked network rapidly [30]. The photocuring process of AESO is faster than that of ASO, as revealed by an onset of polymerization of at less than 2 s compared to the 5 s required for the ASO polymeric precursor. The slower kinetic for ASO-L than AESO-L is also revealed by the decrease in the slope at the initial part of the G’ curve, the curing rate being 72 ± 6 kPa/s for AESO-L and 8.6 ± 0.2 kPa/s for ASO-L, and by the higher t_gel_ of ASO-L (7.0 ± 1.4 s) than AESO-L (1.3 ± 0.2 s). A possible explanation for this is related to the multifunctionality of the synthetized oils and the autoacceleration phenomenon [31]. As ^1^H-NMR reveals, the functionality of the AESO polymeric precursor is significantly higher than that that of the ASO polymeric precursor (2.46 acrylic groups per molecule in the case of AESO versus 1.44 for the ASO) due to the higher effect of the autoacceration for AESO than ASO and, therefore, the higher amount of kinetic energy for the acrylated epoxidized oil than the directly acrylated one.

In addition, G’ values after the photopolymerization plateau are directly related to the mechanical stability of crosslinked materials [32]. Here, the results show that the G’ of the AESO-cured polymer is larger than that of ASO, indicating higher mechanical properties for AESO- than ASO-cured materials, as will be explained in the following section.

Photoreactivity was also characterized by measuring the acrylic double bond (C=C) conversion via FTIR. The average percentages of conversion were calculated following the decrease in the peak at 1630 cm^−1^, corresponding to the mentioned C=C group at the pre- and post-cured materials. The obtained values of conversion (in %), respectively, for the initial C=C content are reported in Table 1, while the FTIR spectra can be seen in Figure 6b.

The FTIR spectra of AESO and ASO formulations display very similar peaks to those characteristic of the main chemical groups presented in the soybean oil-based polymer [11]. Similar to the FTIR spectra of the synthetized polymeric precursors, the stretching vibrations of the -OH, C=O, and C-O-C groups are shown at 3450, 1725 and 1160 cm^−1^, respectively. In addition, the characteristic bands of the asymmetric stretching vibrations and deformations of the C–H bond in the –CH_2_– and –CH_3_– groups are identified at 2920, 2850 and 1450 cm^−1^. Further, the characteristic band of the stretching of C=C group is presented in both formulations at the mentioned 1630 cm^−1^. When samples are irradiated, the success of the C=C conversion can be confirmed by the decrease in the corresponding peak and the difference between pre- and post-irradiated samples is easily observed when spectra are zoomed in on. The conversion degrees are 79 ± 3% for the AESO formulation, while the ASO one presents a significantly higher conversion degree of 93 ± 2%. This can be ascribed to the difference in the functionalization of each oil due to the number of double bonds being different per molecule of AESO or ASO, being 2.46 in the case of AESO and 1.44 for ASO. As has been reported by several authors, the increased number of functionalities presented in the photocurable formulations results in a decrease in C=C conversion due to the vitrification of the highly crosslinked network and the concomitant restricted mobility of the remaining acrylates [33]. Anyhow, high conversion degrees above 75% are confirmed for both formulations.

Once photoreactivity has been assessed, the viscosity of both formulations is measured. In stereolithography or other layer-by-layer printing techniques (as DLP), the resin viscosity is a crucial parameter, with low viscosities desired to enable an appropriate coating of the last cured layer and the resin tank with the liquid resin before beginning the illumination process of the subsequent layer [34]. Moreover, in the preparation of UV-curable formulations, low viscosity oligomers are preferred due to their reduced demand for reactive diluents [35]. However, there is not a fixed range of appropriated viscosities for DLP printing, and some authors have highlighted different values depending on the material used, such as 0.50–0.80 Pa·s for biorenewable formulations [29], a maximum of 10 Pa·s for polymeric materials in general [36], or less than 20 Pa·s at a shear rate of 10 to 100 s^−1^ for polymeric composites [37]. In this sense, viscosities of AESO-L and ASO-L formulations were characterized at room temperature and in the range of 1000 to 0.01 s^−1^ of shear rate. Figure 6c depicts the viscosity curves thus obtained, and Table 1 includes the viscosity values of both materials at a shear rate of 1 s^−1^.

The acrylated soybean oil-based formulations were found to be Newtonian. This can be ascribed to the fact that near-constant viscosity values were showed in the shear rate range tested, as occurs for other natural oils [38]. Particularly, the viscosity of AESO-L is significantly higher (one order of magnitude) than the one of ASO-L, showing values of 11.8 ± 0.6 Pa·s and 1.66 ± 0.04 Pa·s, respectively, at shear rate of 1 s^−1^. In general, there are two major factors that affect the viscosity of acrylated natural oils: molecular weight and H-bonding through OH groups. The viscosity will be higher when the molecular weight increases and when more -OH groups are presented in the formulation [35]. Thus, the higher viscosity of AESO than ASO is justified due to the fact that AESO polymeric precursor presented a higher acrylic functionalization than ASO, which indicates a high molecular weight [35,39,40]. Further, as it is expected, the AESO presents -OH groups in the structure due to the reaction mechanism that consists in the opening of the epoxy rings of ESO and the formation of pendant -OH groups along the double-bond-reacted triglyceride, while ASO is formed via the direct acrylation of SO and OH groups are not formed (also revealed in the FTIR spectra of the polymeric precursors included in the previous section).

### 3.4. Characterization of Solid Samples

**Figure 7:**
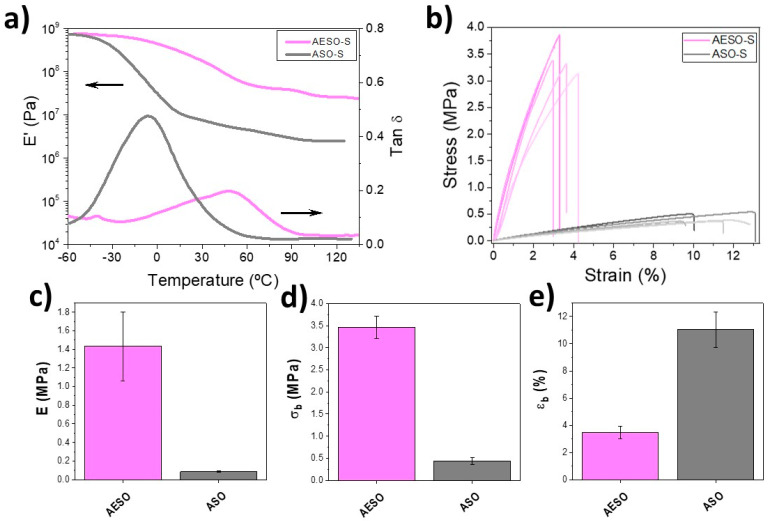
E′ and tan δ curves from dynamic mechanical thermal analysis, arrows indicate the axes of each data (**a**), stress–strain curves, (**b**), Young modulus or E (**c**), strain at break or ε_b_ (**d**) and stress at break or σ_b_ (**e**) of AESO-S and ASO-S samples.

The characterization of solid samples (AESO-S and ASO-S) started with Dynamic Mechanical Thermal Analysis (DMTA), which was employed to fully analyze their thermal and viscoelastic properties. The storage (E′) and loss (E″) moduli were obtained, as well as the ratio between of them named tan *δ* (tan δ = E″/E′). Thus, both E′ and tan δ curves in front of temperature are plotted in Figure 7a, and the storage modulus at the rubbery plateau (E′_R_), the glass transition temperature of each material (T_g_), calculated as the peak on the tan δ curve, and the apparent crosslinking density (ν_c_), obtained as explained in “Section 2”, are detailed in Table 1.

It is possible to observe that both curves present similar shapes. They are characterized by a decrease in the storage modulus when temperature increases and the appearance of a peak in the tan δ plot that corresponds to the decay of the E′. This single E′ step decrease changes from 680 MPa to 40 MPa in AESO-S sample and to 2.6 MPa in ASO-S, corresponding to the transition between the glassy and rubbery states (glass transition) of both materials [41]. Attending to the temperature at which this glass transition occurs, that is, observing the temperature at which the tan δ peak appears, a significantly difference between the acrylated epoxidized and the directly acrylated materials is presented. The T_g_ of AESO-S is located at a higher temperature (around 48 °C) than the one of ASO-S, which appears at −6 °C. Further, the peak is particularly broader for AESO-S than ASO-S, suggesting the existence of a more heterogeneous network for directly acrylated than acrylated epoxidized material [42].

As it is described in the experimental part, the modulus at a rubbery sate (E′_R_) can be related to the crosslinking density (ν_c_) of the polymer networks. Thus, the calculated ν_c_ of AESO-S is significantly higher (2.58 mmol·cm^–3^) than the one of ASO-S (0.26 mmol·cm^–3^). Attending to the results of photopolymerization process, a higher double bond conversion for ASO-S than AESO-S was obtained. This, in theory, should indicate a higher crosslinking degree of directly acrylated material than that for the epoxidized acrylated one [43]. However, the functionality of AESO was estimated as 2.46 acrylic groups per molecule, while for ASO polymeric precursor it was 1.44. In this sense, a conversion of around 80% for AESO supposes a higher number of double bonds converted than a conversion of around 90% of ASO. Therefore, the number of crosslinking points, and hence, the crosslinking density, is higher for AESO than ASO. This effect of crosslinking density also explains the difference observed in the T_g_ of both materials, as occurs in other soybean oil-based materials [44].

The mechanical characterization of AESO-S and ASO-S samples was carried out via stress–strain measurements. Figure 7b reports the obtained curves and the main parameters, that is, Young’s modulus (E) and, the strain (ε_b_), and the stress (σ_b_) at break are plotted in Figure 7c–e. These values are summarized in Table 1.

As can be clearly seen, AESO-S presents a typical stress–strain curves of thermoset brittle materials, characterized by a continued linear increase in the stress with the increase in the strain and the sample break before reaching the yield point at a low value of strain [45]. This indicates the rigid characteristics of the AESO-S material. In the case of ASO-S, a similar behaviour is obtained, even if it shows a larger strain at break and a lower Young modulus. The AESO-S sample presents an E value of around 1.433 ± 0.370 MPa. This is significantly higher (around 20 times more) than the E value obtained for ASO-S, which is 0.085 ± 0.007 MPa. In the case of parameters at break, σ_b_ varies from 3.46 ± 0.25 MPa to 0.43 ± 0.07 MPa and ε_b_ changes from 3.44 ± 0.46% to 11.04 ± 1.31% when soybean oil is directly acrylated.

Typically, the mechanical properties of natural-based oil-cured films are dependent on the inherent chemical composition and the crosslinking density of the oil presented. When photocured polymers are considered, the increase in grafting number is an essential parameter to be considered as the functionality of the oil directly depends on it [46]. Thus, when functionality increases, the number of crosslinking points increases helping this to the increase in the rigidity of the cured films and enhancing the mechanical properties of photocrosslinked materials [47]. In this case, as NMR results reveal, the functionality of AESO is higher than that of ASO, which the subsequent increase in the crosslinking degree as DMA results indicate. Therefore, AESO-S presents a more rigid structure than ASO-S. This is characterized by higher E and σ_b_ values, as well as by a lower ε_b_.

**Figure 8:**
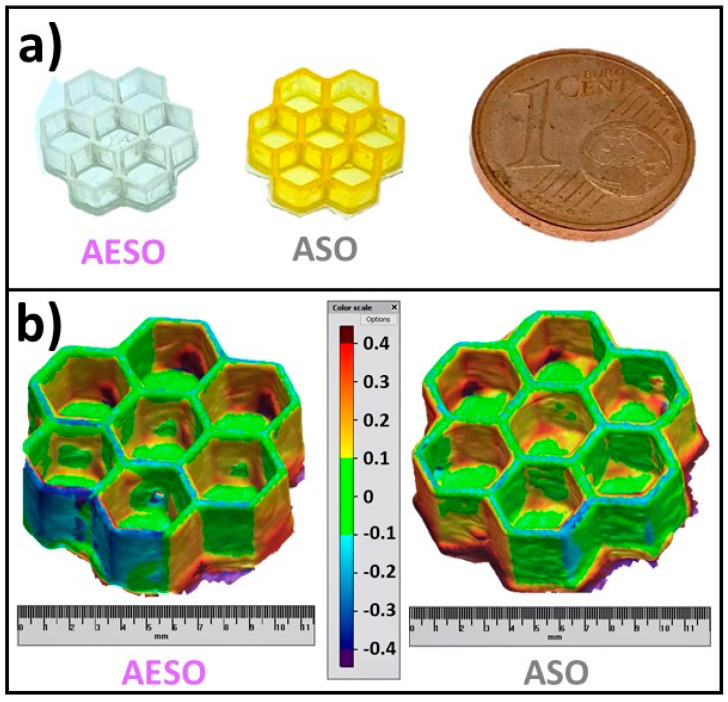
Photographs of honeycomb printed structures for AESO and ASO materials (**a**) and 3D image obtained with 3D scanner with the evaluation of the displacement between the CAD project and the real device for same printed structures (**b**).

Having confirmed the good reactivity and the appropriate viscosities of both soybean oil-based materials for DLP printing, the formulations are successfully 3D-printed. To evaluate the resolution and the capabilities of both AESO-L and ASO-L materials in DLP, a hexagonal honeycomb 3D form is printed. The 3D-printed structures are characterized via a 3D scanner; Figure 8 shows the real photos of structures printed using both soybean oil-based materials, as well as an analysis of them obtained using the scanner.

In previous probes, the different printing parameters were properly adjusted depending on the material employed. As the AESO-based formulation presents a higher viscosity compared to ASO-based one, the working temperature for 3D printer slightly increases from room temperature to 40 °C in order to facilitate the movement of the formulation during the layer-by-layer process. Further, different parameters such as exposure time, platform approximation velocity, or light intensity, were adjusted to obtain 3D structures with a consistent form (self-supporting structures) in short periods of time. The methods did not prioritize prevention over curing and had the highest possible quality. Thus, in a honeycomb structure, great results are obtained for both AESO and ASO materials, as Figure 8a depicts. In the case of an AESO-printed structure, an exposure time of 2 s per layer is enough to properly obtain the whole structure. Meantime, for ASO material, an exposure time of 4 s is needed (light intensity of 35 mW/cm^2^ for both materials). This is in concordance with the photorheology results that demonstrate a lowest reactivity for the ASO formulation than the AESO one. In terms of the platform approximation velocity, the lowest viscosity of ASO material than AESO allows for the use of a higher velocity, reducing the total time needed for printing. In this sense, the exposure time of both parameters, and the platform approximation velocity present the opposite effect, and the total times required to print the honeycomb structures are similar for both materials. Similarly, other photocurable resins, printed using the same printer as this study, also use the same printing parameters as the ones employed here [48], indicating the good ability of these materials to be used as DLP resins.

Attending to the resolution and the quality of the printed structures, both materials present a good similar appearance. Both AESO-S and ASO-S 3D-printed structures are investigated via a 3D scanner in order to evaluate their CAD fidelity. Figure 8b presents the comparison between the 3D image and the CAD virtual project. As can be seen, both printed structures are colored in green, indicating the great resolution and the fidelity of the project for both of them. The green color represents a displacement at about ± 0.1 mm and yellow and light blue colors are about ±0.2 mm. These results are to those obtained on other soybean oil-based materials printed via DLP [49].

## 4. Conclusions

In summary, acrylated epoxidized soybean oil (AESO) and acrylated soybean oil (ASO) have been successfully obtained from epoxidized soybean oil (ESO) and soybean oil (SO), respectively. In the case of AESO, five catalysts have been studied. In particular, we examined chromium (III) 2-ethyl hexanoate (Cr(EH)3-MO) in 70% in mineral oil, the one with higher reaction yield according to the epoxide conversion and acidity index. For ASO, the reaction has been performed by employing the boron trifluoride at 48% in diethyl ether (BF3OEt2) as an efficient Lewis acid, obtaining the product with a lower rate of acrylate molecules per triglyceride than AESO. Both AESO and ASO structures have been confirmed via the 1H-NMR technique. Additionally, oils have been mixed with an appropriate quantity of BAPO photoinitiator and the resulting formulations have been analyzed in terms of 3D printability via photorheology and viscosity measurements. Both AESO and ASO have been demonstrated to be suitable materials for DLP printing and their printability has been studied by analyzing the quality of 3D-printed part by means of a 3D scanner that indicates an accuracy of ±0.1 mm for both oils. The AESO photocured materials showed higher glass transition temperature, crosslinking density and Young’s modulus than ASO, which can be ascribed to the lower acrylate group rate presented. In conclusion, this work demonstrates the possibility of using biobased materials as photocurable inks for 3D printing and the possibility of obtaining them via one-step reactions from the original vegetable resource.

## Figures and Tables

**Table 1:** Main properties for AESO and ASO liquid and solid materials.

Property	AESO	ASO
t_gel_ (s)	1.3 ± 0.2	7.0 ± 1.4
ΔG′/Δt (kPa/s)	72 ± 6	8.6 ± 0.2
Conversion (%)	79 ± 3	93 ± 2
$\mathrm{Viscosity}\;\mathrm{at}\;\dot{\gamma }=1\;s^{-1}$ (Pa·s)	11.8 ± 0.6	1.66 ± 0.04
E′_R_ (MPa)	40	2.6
T_g_ (°C)	48	−6
ν_c_ (mmol·cm^–3^)	2.58	0.26
∆V(%)	5.0 ± 0.2	4.3 ± 0.4
E (MPa)	1.433 ± 0.370	0.085 ± 0.007
σ_b_ (MPa)	3.46 ± 0.25	0.43 ± 0.07
ε_b_ (%)	3.44 ± 0.46	11.04 ± 1.31

## Data Availability

The raw/processed data required to reproduce these findings cannot be shared at this time as the data also forms part of an ongoing study.
